# The roles of transforming growth factor-β, Wnt, Notch and hypoxia on liver progenitor cells in primary liver tumours

**DOI:** 10.3892/ijo.2014.2286

**Published:** 2014-02-03

**Authors:** ELIENE BOGAERTS, FEMKE HEINDRYCKX, YVES-PAUL VANDEWYNCKEL, LEO A. VAN GRUNSVEN, HANS VAN VLIERBERGHE

**Affiliations:** 1 Department of Gastroenterology and Hepatology, 1K12, Ghent University Hospital, 9000 Gent, Belgium;; 2 Department of Medical Biochemistry and Microbiology, Uppsala University, 751 23 Uppsala, Sweden;; 3 Department of Cell Biology, Liver Cell Biology Lab, Vrije Universiteit Brussel, 1090 Brussels, Belgium

**Keywords:** hepatic cancer, liver progenitor cell, cancer stem cells, Wnt receptors, TGF-β, Notch receptors, hypoxia inducible factor 1

## Abstract

Primary liver tumours have a high incidence and mortality. The most important forms are hepatocellular carcinoma and intrahepatic cholangiocarcinoma, both can occur together in the mixed phenotype hepatocellular-cholangiocarcinoma. Liver progenitor cells (LPCs) are bipotential stem cells activated in case of severe liver damage and are capable of forming both cholangiocytes and hepatocytes. Possibly, alterations in Wnt, transforming growth factor-β, Notch and hypoxia pathways in these LPCs can cause them to give rise to cancer stem cells, capable of driving tumourigenesis. In this review, we summarize and discuss current knowledge on the role of these pathways in LPC activation and differentiation during hepatocarcinogenesis.

## Contents

1. Introduction
2. Liver progenitor cells
3. Liver progenitor cells in hepatic carcinogenesis
4. Role of hypoxia in hepatic carcinogenesis and progenitor cell activation
5. Conclusion

## 1. Introduction

Liver cancer is one of the most frequently diagnosed cancers worldwide. Despite efforts made, these tumours are often detected in an advanced stage, making liver cancer the third most deadly cancer worldwide (1). The most important types of primary liver cancer are hepatocellular carcinoma (HCC) and intrahepatic cholangiocarcinoma (ICC). HCC often develops in a background of chronic liver disease caused by chronic alcohol abuse, viral hepatitis or non-alcoholic steatohepatitis, while less is known on potential risk factors for ICC. Both primary tumours can be found together in combined hepatocellular-cholangiocarcinoma (CHC), which is characterised by a worse prognosis than HCC or ICC (2,3). There are several curative therapeutic options for primary liver tumours including resection, transplantation and radiofrequency ablation. However, more often than not, these tumours are detected in late stages. At this point, existing therapies including anti-angiogenic compounds such as sorafenib, and transarterial chemoembolisation (TACE) (4), mainly aim to slow down tumour growth and increase survival. Unfortunately, these treatment strategies still hold various serious adverse effects and therapy resistance, relapse and metastasis remain a real threat (4–6). Importantly, anti-angiogenic treatment also sometimes causes increased local invasion and metastasis, worsening tumour progression (5). Finally, a phenotypic switch from HCC to CHC has been reported after both TACE and increased hypoxia inducible factor (HIF) stabilisation in a mouse model for HCC (6,7).

Cancer stem cells (CSC) are cancer cells that possess stem cell characteristics such as the ability to differentiate to all cell types found in a particular cancer sample and are associated with relapse and metastasis (8,9). Recently, interest has grown in the existence of liver CSC with a liver progenitor cell (LPC) gene signature, LPCs are triggered during severe acute or chronic liver injury, during which proliferation of mature hepatocytes is inhibited (10). LPC-progeny can express hepatocyte- or cholangiocyte-specific lineage markers and experimentally have been proven to differentiate into either of these cell types (11–13).

Possibly, adverse effects often seen following treatment could be caused by survival and adaptation of LPC derived CSC. This would indicate that LPCs could not only play a role in tumour initiation, but also in progression and therapy resistance (14–17).

This review briefly summarizes the current knowledge on signalling pathways acting in primary liver tumour biology, specifically their involvement in LPC activation and proliferation, as well as a possible relation between LPCs and CSCs.

## 2. Liver progenitor cells

In case of severe hepatic damage, such as in elaborate chronic liver injury, when proliferation of hepatocytes and/or cholangiocytes alone is insufficient to restore the liver mass and function, liver progenitor cells (LPCs) are stimulated to proliferate and replace the damaged cell types (12). Even though LPCs can most commonly be found in the canals of Hering (18,19), several other possible locations have been described: intralobular bile ducts, peri-ductal cells and peribiliary hepatocytes (20). Possibly, the LPC niche also consists of other actors in liver damage, such as hepatic stellate cells and Kupffer cells (21–23). Differential interaction with these cells could account for the different observations concerning LPC location and factors involved in their activation in various models of liver injury (19,22,23).

The most commonly used markers for identification of LPCs, or determination of cells with LPC-like characteristics are Prominin 1 (CD133), epithelial cell adhesion molecule (EpCAM), α-fetoprotein (AFP), and (cyto-) keratin 19 (CK19). However, many other stem cell, hepatic and cholangiocytic markers are used to characterize LPCs (Table I) (24–26).

Although the existence of LPCs and their role in liver injury is generally accepted, and a broad range of markers is being used to identify and/or isolate these cells from livers (13,19,27–29), researchers have not yet agreed on a precise set of markers defining the LPC population, therefore filtering out the identity of the ‘true progenitor cell’, remains a challenge.

## 3. Liver progenitor cells in hepatic carcinogenesis

Several studies have shown that cells with LPC characteristics are part of the tumour niche in primary liver tumours (30–32). Because of their multipotent characteristics there probably is a role for LPCs in HCC and ICC formation, however, due to the dual hepatocytic and cholangiocytic origin, it is the CHC that is generally presumed to be a progenitor derived tumour (30,33).

Currently, there are two major hypotheses on how stem cells influence tumour formation. Firstly, the clonal evolution model, which presumes that a single cell acquires random mutations and gives rise to a group of identical tumour cells, each with equal potential to generate a tumour. Secondly, the cancer stem cell theory proposes that a tumour consists of a heterozygous cell population, where only certain cells are able to self-renew and differentiate (9).

Over the years, CSC have been shown to play a role in the development of certain forms of leukaemia and glioblastoma, as well as in several solid tumours such as breast, gastric and colon cancer (15,24,34) and are now being extensively studied in hepatocarcinogenesis (15,24).

The predisposition of primary liver tumours to develop in a background of chronic liver disease in which there is an increased proliferation of progenitor cells (2,7) increases the likelihood of progenitor cells accumulating and stabilising enough mutations to obtain a cancerous phenotype. It may thus be possible for LPCs to transform into (hepatic) cancer stem cells and grow into primary liver tumours (15,24).

So far, several pathways have been shown to mediate LPC activation, proliferation and/or differentiation. The balance between Wnt and Notch signalling has been proposed to be crucial for determination of the LPC cell fate. Activation of the Notch pathway is essential for biliary differentiation, as shown by several *in vivo* and *in vitro* experiments (35,36). Moreover, in case of hepatocyte injury, activation of the canonical Wnt pathway, probably prevents activation of the Notch pathway, thus pushing LPC differentiation towards hepatocytes (35,36). Also, interaction between tumour cells and the extracellular matrix (ECM) is shown to be essential for tumour progression, invasion and metastasis, transforming growth factor-β (TGF-β)-mediated epithelial mesenchymal transition (EMT) plays an important role in this interaction (37). Recently TGF-β signalling has also been linked to the presence of LPCs in hepatocarcinogenesis (38).

**Figure 1.**
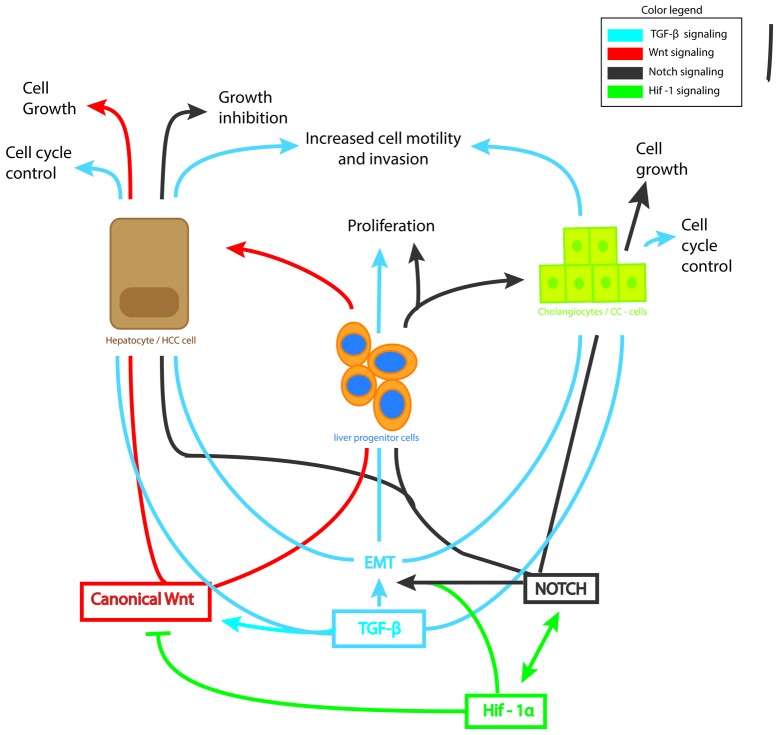
Schematic representation of the role of Wnt, Notch, TGF-β and Hif-1α signalling in hepatocytes, cholangiocytes and liver progenitor cells in hepatocarcinogenesis. The cell growth promoting effects of the Wnt and Notch pathways on hepatocytes and cholangiocytes, respectively, as well as their differential role on liver progenitor cells. The complicated dual role of TGF-β as guardian of cell cycle control, as well as its tumour promoting and invasion and metastasis inducing potential in all cell types is visualised. Finally, the complex interactions between these three pathways, and the possible influence of the HIF-1 pathway is presented.

The Notch, Wnt and TGF-β pathways are also well known to be involved in many tumourigenic processes. In this review we will focus on these three pathways and discuss their role in hepatocarcinogenesis, with special attention to their potential involvement in LPC and/or CSC-mediated tumour initiation and progression (Fig. 1).

### Wnt/β-catenin pathway

The canonical Wnt signalling pathway directs essential cell regulatory mechanisms such as cell proliferation and cell polarity, but also plays an important role during embryonic development (39–41).

A key player in the canonical Wnt signalling pathway is β-catenin, which also plays a crucial role in intracellular junctions by forming a receptor complex with epithelial cadherin (E-cadherin) (39). Upon binding of Wnt to its receptor Frizzled, β-catenin switches from being part of a destruction complex to the formation of a ‘Wnt-signalosome’ that prevents β-catenin degradation. This allows the latter to migrate to the nucleus where it binds to the T-cell factor/lymphoid enhancer factor and induces transcriptional activation of Wnt-responsive genes (39,42). This β-catenin signalling has been shown to be necessary for mouse LPC activation upon injury in rodents (43) and to regulate the hepatocytic specification of LPCs (35).

In HCC cell lines, activation of the Wnt/β-catenin signalling pathway not only increases EpCAM accumulation in both the cytoplasm and the nucleus (42), but also increases the EpCAM^+^AFP^+^ and the oval cell marker 6 (OV6)^+^ population. These represent cell populations with strong LPC features which also demonstrate tumourigenic and invasive capacities (41,44). Canonical signalling probably also plays a role in chemoresistance, which is strongly linked to LPC proliferation (45,46), as shown by the increased EpCAM expression in patients with reduced sensitivity to interferon α/5-fluorouracil combination therapy (46). In addition, blocking the Wnt/β-catenin pathway not only inhibits HCC cell growth (42), but also diminishes chemoresistant OV6^+^ colonies (41).

Interestingly, canonical and non-canonical Wnt pathways seem to have opposing effects on tumour growth (47–49). The canonical pathway (mediated by Wnt1-3) mediates growth and regeneration and is reported activated in well differentiated HCC cells while it is repressed in poorly differentiated HCC cell lines (41,43,49). Oppositely, activating the non-canonical pathway (including Wnt5a and 11) has been shown to inhibit HCC and ICC growth (47–49), possibly by antagonizing the canonical pathway, and promoting cell motility and invasion (49). This could indicate an important role in the growth and migration pattern of the tumour, caused by interaction between these two pathways during hepatocarcinogenesis.

### Transforming growth factor-β pathway

TGF-β is involved in various cellular functions, such as cell growth, differentiation and apoptosis, both in adult as well as in embryonic stages (50). Binding of TGF-β to its receptor results in phosphorylation of the receptor eventually followed by the translocation of Smad proteins (Smad2/3) to the nucleus in a complex with Smad4 (coSmad), where they can regulate transcription by binding to Smad-binding elements in co-operation with a plethora of Smad interacting proteins (51,52). However, TGF-β also uses non-Smad signaling pathways such as the phosphoinositide 3-kinase/Akt/mTOR pathway, the p38 and Jun N-terminal kinase/mitogen-activated protein kinase pathway to transduce its signals (53). In addition to these non-canonical pathways, TGF-β signalling is regulated at many levels by processes such as endocytosis of the receptor complex, or by molecules like inhibitory Smads6/7 and the bio-activity of the ligands through proteolytic cleavage by their protease (mainly furin) (51).

Like its regulation, the role of TGF-β in tumour formation is rather complicated. In healthy tissue, it acts as a tumour suppressor controlling the cell cycle, inducing apoptosis and regulating autophagy. During tumourigenesis, cells switch their response to TGF-β, making it a potent inducer of cell motility, invasion and metastasis, as well as guardian of stem cell maintenance (54). In liver carcinogenesis, TGF-β has been shown to have both tumour suppressing and promoting effects (24,50) and its expression is decreased in early, while increased in later stages of tumourigenesis (24,55,56).

TGF-β signalling is also a master regulator of initiating and maintaining EMT, the process directing cancer cells towards invasion and metastasis (37). In HCC cells, inhibition of TGF-β has been reported to upregulate epithelial-cadherin (E-cadherin) and thereby lower migration and invasion potential (57). However, in human fetal hepatocytes (cells carrying progenitor cell features, like EpCAM and CK19 as well as hepatoblast features like AFP), TGF-β even induces apoptotic, growth inhibitory signals, as well as pro-invasive, mesenchymal characteristics such as neuronal cadherin, Snail and vimentin (57). What is more, during EMT, TGF-β signalling results in dissociation of β-catenin from the E-cadherin/ β-catenin membrane complex resulting in cytoplasmatic and nuclear accumulation of β-catenin and subsequent activation of the Wnt pathway (58). Possibly, this upregulation of the Wnt pathway, due to TGF-β dysregulation causes a larger population of activated LPCs in HCC patients (59) and in mice following partial hepatectomy (60). Furthermore, in patients, high nuclear β-catenin accumulation is correlated with higher vascular invasion grades and increased recurrence after transplantation (59).

These data suggest an important, but contradictory role for TGF-β signalling in hepatocarcinogenesis, possibly regulating the activation and differentiation of LPCs, through regulation of the Wnt-signalling pathway. Because of the important role of TGF-β in EMT, its regulation is decisive for the invasive and metastatic potential of the tumours.

### Notch pathway

The Notch pathway is important in stem cell self-renewal, differentiation, and plays a special role in the control of many binary cell fate choices in embryonic and adult cells (61). In the liver, Notch signalling promotes differentiation of LPCs towards the cholangiocytic lineage rather than to hepatocytes (62). Furthermore, Notch is involved in several fundamental cell regulatory processes such as proliferation, apoptosis and EMT (61). Binding of Delta or Jagged ligand to the Notch receptor, causes cleavage of the extracellular C-terminal peptide. Notch intracellular domain (NICD) is then cleaved by γ-secretase, releasing it into the cytoplasm so it can migrate to the nucleus, bind to CSL, recruit co-activators such as mastermind-like, and induce Notch-dependent gene transcription. The two major targets are the Hairy and Hes-related repressor protein families of transcription factors (61,63).

Like the Wnt and TGF-β pathway, aberrant Notch signalling is well described in many different kinds of cancer, such as breast, lung, colorectal, pancreatic and hepatic cancer (24,63). However, deregulation of the Notch pathway has been described as both oncogenic and tumour suppressive, depending on tissue type and circumstances (63–65).

For example, the effect of Notch signalling on hepatocarcinogenesis can be determined by its effect on several players in cell cycle control such as p53 (65), cyclin-A, -D1 and -E (64). Induction of p53 in HepG2 cells, leads to an increased expression of NICD and downregulation of the cells proliferative capacity, but not the other way around. Moreover, in cells expressing mutant p53, not able to induce NICD upregulation, administration of recombinant NICD protein did cause reduced proliferation (65).

In a different HCC cell line, SMMC7721, NICD overexpression by retroviral transfection did cause increased p53 levels, as well as decreased levels of proteins involved in cell cycle control, like phosphorylated forms of the retinoblastoma protein, thus also causing inhibition of growth and proliferation (64). Unfortunately neither of these studies investigated the LPC properties of the used cells, before nor after p53 or NICD induction.

In accordance, Notch pathway inhibition by DAPT (γ-secretase inhibitor) in adult mice after conditional deletion of retinoblastoma protein family genes in the liver, which causes proliferation of the progenitor compartment, resulted in an increased number of HCC nodules (66). Also, over-activation of NICD inhibits cell proliferation in tumour cell lines derived from these retinoblastoma-deficient mice, but not in HepG2 cells (66). These data suggest a differential role for the Notch pathway in progenitor cells compared to hepatocytes, further supported by recent findings of hepatocyte-specific NICD overexpression causing development of HCC with 100% penetrance after 12 months (67) and ICC after partial hepatectomy (68).

Finally, Notch signalling has also been related to therapy resistance; Delta-like ligand induced activation of the Notch pathway seems to mediate tumour resistance to anti-angiogenic therapy by activating escape mechanisms in the tumour causing the formation of new vessels circumnavigating the therapy-induced blockage (69,70).

## 4. Role of hypoxia in hepatic carcinogenesis and progenitor cell activation

In the presence of oxygen, HIF is quickly hydroxylated by prolyl hydroxylase domain proteins, causing degradation. However, in hypoxic conditions, shortage of hydroxyl-groups leads to HIF stabilisation and migration to the nucleus where it regulates processes supporting cell survival under hypoxic conditions, for example by increasing (neo)angiogenesis (71). Primary liver tumours, especially HCC, often develop in a background of chronic liver disease, characterised by fibrogenesis, eventually leading to cirrhosis. This process is accompanied by increased hypoxia, caused by sinusoidal capillarisation and formation of fibrotic septa increasing resistance to blood flow and thus decreasing oxygen delivery to liver cells. In addition, the fast growing liver tumours quickly outgrow the existing liver vascularisation, thus creating hypoxic conditions (7,72,73).

Current treatment strategies for advanced stage liver cancer, such as anti-angiogenic treatment or TACE, often aim to deprive the tumour of its blood and nutrient supply (4). However, therapy resistance to TACE and anti-angiogenic treatment has been attributed to induction of hypoxic conditions and activation of HIF (3,7,74), by adversely increasing cancer cell survival and tumour growth.

Recently, a significant increase in stem cell marker expression has been seen *in vitro* after exposure of HCC cultures to hypoxia (75). Possibly, the decreased oxygen levels in tumour cells stimulate dedifferentiation towards a progenitor phenotype. Potentially increased proliferation and altered differentiation of LPCs in HCC also cause the phenotypic switch to CHC in prolyl hydroxylase domain 2 heterozygous mice, which are characterised by increased HIF stabilisation (3,7) and in patients, after receiving TACE treatment (6).

These findings have raised many questions about the future of these therapies, since monotherapies are often insufficient in treatment of HCC and can even induce more aggressive disease. It is of vast importance to consider alternative therapeutic strategies that prevent this massive hypoxic response. For example, a recent study has shown a better outcome in mice with HCC, after treatment with anti-placental growth factor, causing vascular normalisation, instead of blocking neoangiogenesis, and thus causing less hypoxia (3). Also, administration of EF24, could synergistically enhance the antitumour effects of sorafenib, reduce metastasis and overcome sorafenib resistance through inhibiting HIF-1α by sequestering it in the cytoplasm and promoting degradation by upregulating the Von Hippel-Lindau tumour suppressor in five different cell lines and in both xenograft and orthotopic mouse models for HCC (76).

Possibly, a HIF-dependent alterations to the Wnt, Notch and/or TGF-β pathways are responsible for the observed reaction of tumour tissue to hypoxia inducing therapies. Both *in vitro* and *in vivo* experiments have shown crosstalk between the Wnt and HIF pathways, depletion of β-catenin resulted in more severe hepatic injury in a mouse model for liver perfusion while an increased Wnt signalisation resulted in a marked decrease of hepatic injury compared to control (77). In this study, Wnt1 overexpression resulted in a significant higher response of HIF sensitive genes and HIF1α protein levels, While β-catenin/T-cell factor target gene expression was significantly reduced after ischemia, without a decrease in total β-catenin. The observation was further supported in HCC cells *in vitro*, where a direct interaction between HIF1α and β-catenin was shown, enhancing HIF1α signaling and driving EMT (78). Thus, in hypoxic conditions, HIF1α competes with the lymphoid enhancer factor for binding of transcriptional activator β-catenin inhibiting the canonical Wnt pathway responsible for hepatocyte proliferation and instead promoting adaptation, survival and EMT through HIF signalling (77,78). This further demonstrates the potency for intratumoural hypoxia to push LPC differentiation towards a more aggressive, therapy-resistant cancerous offspring.

Furthermore, the epithelial mesenchymal transition of hepatocytes could also contribute to dedifferentiation of hepatocytes towards a stem/progenitor-like phenotype as seen *in vitro* (79). EMT in hypoxic conditions is probably accomplished by HIF mediated activation of the TGF-β pathway (80,81). Next to the β-catenin induced intensification, Notch1 signaling has been shown not only essential for HIF and snail mediated EMT (82,83), but also capable of inducing EMT in normoxic conditions by directly targeting Snail in breast cancer cell lines (83). However, in an HCC cell line a direct interaction between NICD and Snail in the cytoplasm has been shown to result in ubiquitinylation and degradation of Snail (84), again, showing the complex nature of these cell-type specific interactions.

## 5. Conclusion

Despite the increase in scientific interest, the role of LPCs in cancer progression is still unclear. These bipotential progenitor cells could shift to a cancerous phenotype and give rise to HCC, ICC and CHC, and not only regulating tumour initiation and growth, but also the invasive and metastatic potential. Likely, specific interactions between several pathways involved in regulation of LPCs can be modulated by intrinsic as well as extrinsic factors and is capable of driving tumourigenesis and determining its phenotype. Of the 3 main liver tumours potentially derived from LPCs, CHC is most suitable to study the role of bipotential cells during tumour formation, since it consists of both hepatocyte- and cholangiocyte-like cells (85). We discussed a role for altered regulation of Notch, Wnt, HIF and TGF-β signalling in primary liver tumour development. Interactions between these pathways could possibly force a group of progenitor or cancer stem cells to behave differently, causing a tumour to exhibit both HCC and ICC-like characteristics.

There is also a potential role for hypoxia in the determination of cell fate in LPCs, possibly not only by triggering conversion of its tumourigenic offspring to a more malignant, mixed phenotype (6,7), but also by inducing therapy resistance (69,86). As discussed here, the major target of altered signalling could be the EMT, a major process in malignant conversion, provoking hepatocytes to exhibit more stem/progenitor-like features and thus increasing the pool of cancer cells with an LPC signature.

These findings are of particular interest when using therapies altering the signalling of one or more of these pathways, triggering changes which could potentially lead to more aggressive tumours. More specifically, inhibiting the involvement of the Notch, Wnt or TGF-β pathway could be the key to altering the massive response to hypoxia and would allow us to reduce the adverse effects so often caused by hypoxia-inducing therapy.

## Figures and Tables

**Table I.** Selection of LPC markers and their potential role in hepatocarcinogenesis.

Abbreviation	Full name	Role in HCC and/or CC development
CK7	(cyto) keratin 7	Increased expression of these cholangiocytic markers in primary liver
CK19	(cyto) keratin 19	tumours indicate poor prognosis (16,87)
ALB	Albumin	Hepatocyte-specific marker, upregulated in ICC, compared to other cholangiocellular tumours like extrahepatic cholangiocarcinoma (88,89)
OPN	Osteopontin	Restricted to cholangiocytes lining the canals of Hering, good LPC marker for lineage studies (12)
OCT4/Pou5f1	Octamere binding transcription factor/Pou domain class 5, transcription factor 1	Embryonic transcription factor involved in stem cell self-renewal. Possible prognostic marker for HCC, and upregulated in chemoresistant liver cancer cells (90)
AFP	α-fetoprotein	Fetal serum protein, often but not always re-expressed in HCC and CHC (89,91)
LIF	Leukemia inhibitory factor	Cells are pushed to differentiate during decreased LIF levels. LIF is elevated in LPCs and known to induce acute phase proteins in hepatocytes (92).
Sox 9	SRY-related HMG box transcription factor 9	Transcription factor involved in cholangiocyte-specific development (93)
CD133	Prominin 1	Cancer stem cell marker, upregulated in most primary liver cancers. Associated with more aggressive phenotype and therapy resistance (94–96)
CD34	CD34 antigen	Cancer cell marker mainly expressed in early hematopoietic cells.
CD44	CD44 antigen	Upregulated in most primary liver cancers, regulation associated with more aggressive phenotype and treatment resistance (96)
CD56/NCAM	Neural cell adhesion molecule	Shift from E-cadherin to NCAM expression indicates epithelial mesenchymal transition
CD117	c-Kit	Proto-oncogene, upregulation due to mutation occurs in many tumours. C-Kit inhibition is also reported to slow LPC expansion and tumour formation in rodents (97)

## Abbreviations:

HCC: hepatocellular carcinoma;
ICC: intrahepatic cholangiocarcinoma;
CHC: hepatocellular-cholangiocarcinoma;
TACE: transarterial chemoembolisation;
HIF: hypoxia inducible factor;
CSC: cancer stem cell;
LPC: liver progenitor cell;
TGF-β: transforming growth factor-β;
CD133: prominin 1;
EpCAM: epithelial cell adhesion molecule;
AFP: α-fetoprotein;
CK19: cytokeratin 19;
ECM: extracellular matrix;
EMT: epithelial mesenchymal transition;
OV6: oval cell marker 6;
NICD: Notch intracellular domain
